# Supplementary material for: A chemical-genetic system to rapidly inhibit the PP2A-B56 phosphatase reveals a role at metaphase kinetochores
Source: Nat Commun. 2025 Mar 29;16:3069. doi: 10.1038/s41467-025-58185-8 (PMC11954910; doi:10.1038/s41467-025-58185-8)
Supplement: Supplementary file 1 — Supplementary Information [file 41467_2025_58185_MOESM1_ESM.pdf]

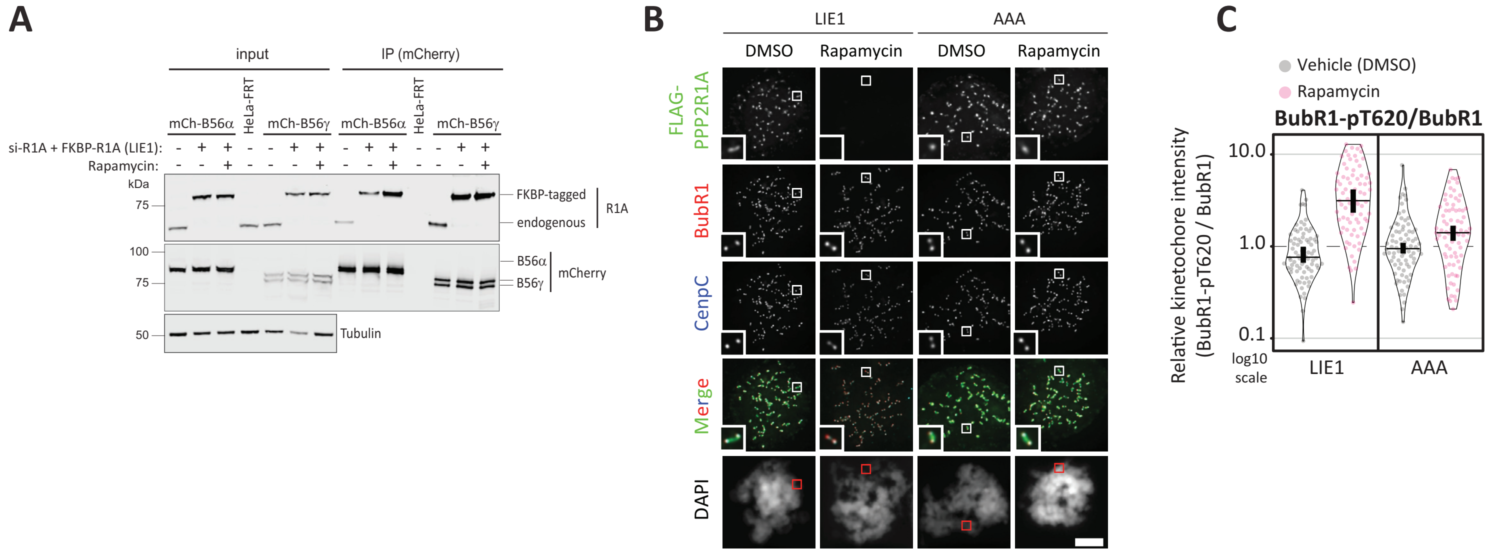

Supplementary Figure 1 (related to Figure 1). A. Immunoblot of endogenous and FKBP-tagged R1A protein following mCherry-B56 immunoprecipitation from nocodazole-arrested HeLa FRT cells expressing either mCherry-B56α or mCherry-B56γ, with/without directSLiM-LIE1 expression, -/+ rapamycin for 30min. **B.** Representative example immunofluorescence images of the BUBR1 kinetochore quantifications shown in Figure 1E. **C.** Relative quantification of the ratio of BUBR1/BUBR1-pT620 within individual LIE or AAA cells treated with DMSO control or rapamycin to 20 min. 80 cells per condition from 4 repeats. Violin plots show the distributions of kinetochore intensities between cells. Source data are provided as a Source Data file. For each violin plot, each dot represents an individual cell, the horizontal line represents the median and the vertical one the 95% CI of the median, which can be used for statistical comparison of different conditions (see Methods).

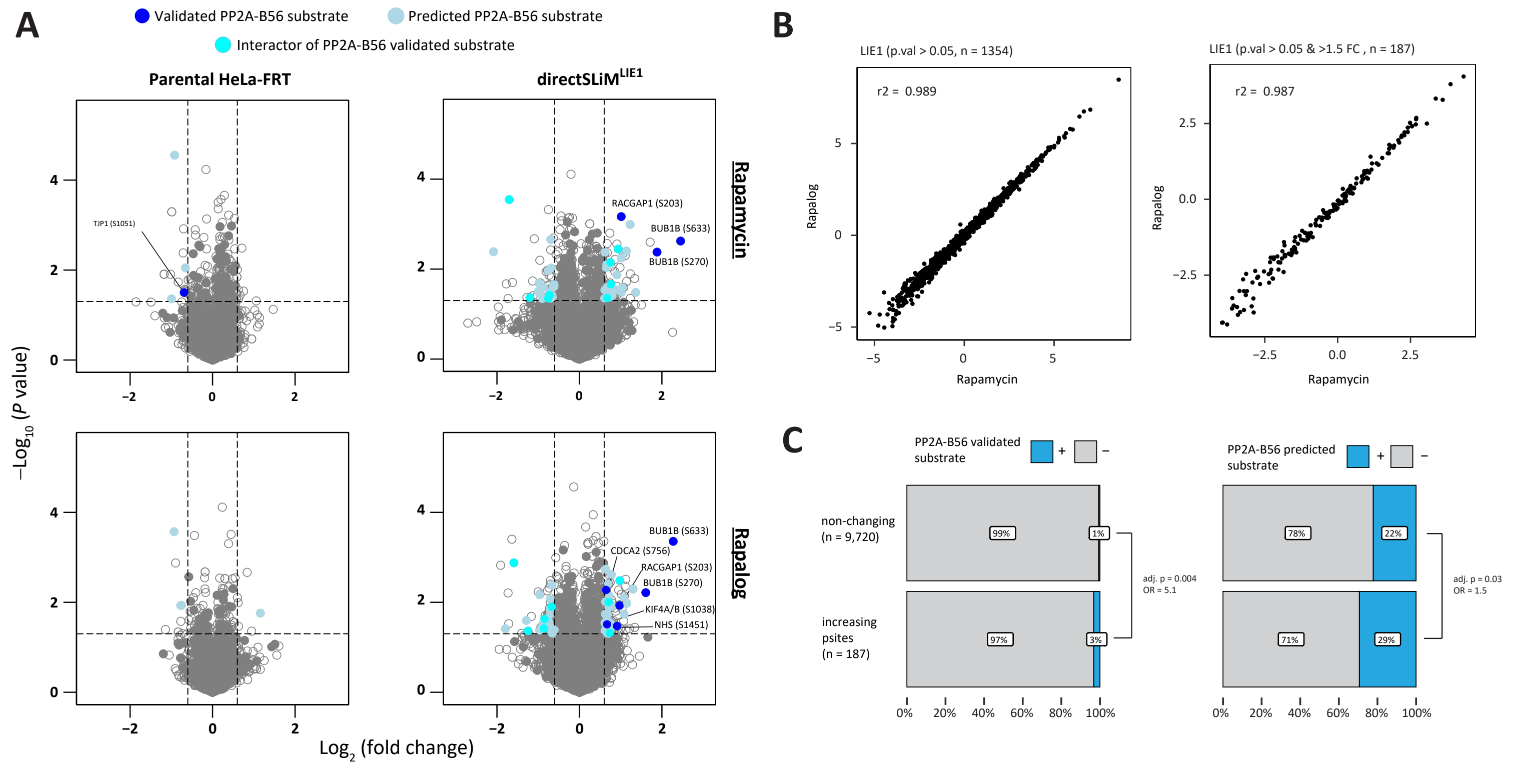

Supplementary Figure 2 (related to Figure 2). A. Volcano plot showing upregulated and downregulated phosphorylation sites after treatment with rapamycin or rapalog in directSLiM^{LIE1} cells. Solid dots are either validated substrates, predicted substrates or interactors of validated PP2A-B56 substrates (see methods for details). Only hits above a -Log10(P value = 0.05) (Two sided Student's t-test) and >1.5-fold change are color coded. **B.** Scatter plot showing correlation of rapamycin and rapalog treatment in all changing sites (left) and in the top changing sites (right) in directSLiM^{LIE1} cells. **C.** Assessing enrichment of PP2A-B56 validated (left) and predicted (right) substrates in proteins with increasing phosphorylation sites (p values and odds ratio reported from Fisher's exact test). Source data are provided as a Source Data file.

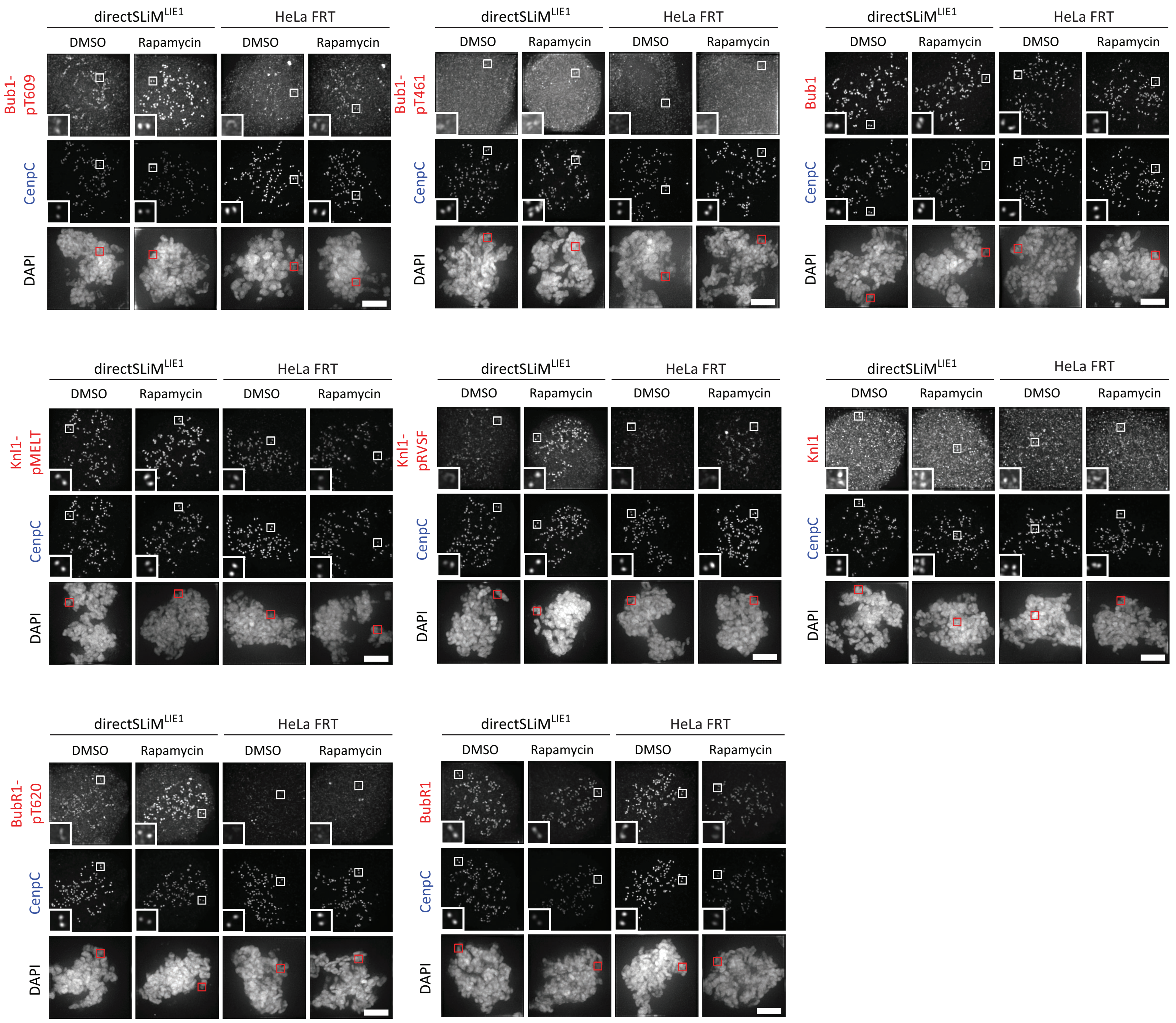

Supplementary Figure 3 (related to Figure 3). Representative example immunofluorescence images of the kinetochore quantifications shown in Figure 3B-C. The insets show magnifications of the outlined regions. Scale bars: 5µm. Inset size: 1.5µm

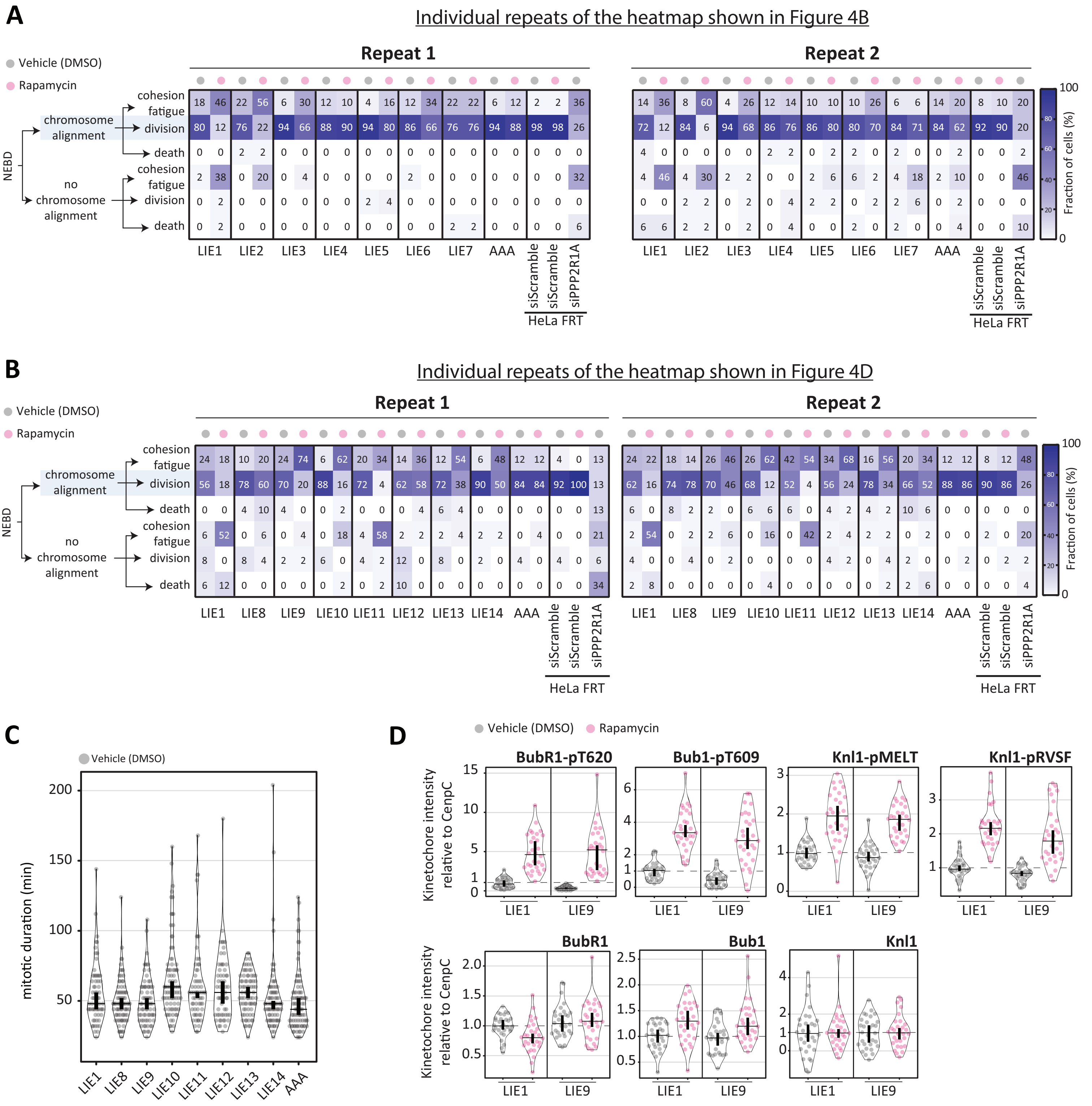

Supplementary Figure 4 (related to Figure 4) A-B. The two heatmaps show the frequencies of the cell fates shown in the 2 experimental repeats from the data in Figure 4B (A) and Figure 4D (B). **C.** Mitotic duration – measured as the time between NEBD and chromosome segregation – of the vehicle-treated cells dividing after nuclear envelope breakdown (NEBD) shown in 4D. Number of cells from 2 experiments: 65 for LIE1, 80 for LIE8, 71 for LIE9, 81 for LIE10, 63 for LIE11, 59 for LIE12, 78 for LIE13, 80 for LIE14, 88 for AAA. **D.** Levels of BubR1-pT620, Bub1-pT609, Knl1-pMELT, Knl1-pRVSF, BubR1, Bub1 and Knl1 at unattached kinetochores, in nocodazole-arrested HeLa FRT cells expressing directSLiMs$^{LIE1/LIE9}$ and treated with vehicle or rapamycin for 20min. Note that the distributions of the vehicle-treated directSLiM^{LIE1} and directSLiM^{LIE9} cells are also shown in Figure 4E. Kinetochore intensities from 30 cells, 3 experiments. Source data are provided as a Source Data file.

Data information: Kinetochore intensities in D are normalized to LIE1 vehicle condition. Violin plots show the distributions of mitotic duration (C) or the distributions of kinetochore intensities between cells (D). For each violin plot, each dot represents an individual cell, the horizontal line represents the median and the vertical one the 95% CI of the median, which can be used for statistical comparison of different conditions (see Materials and Methods).

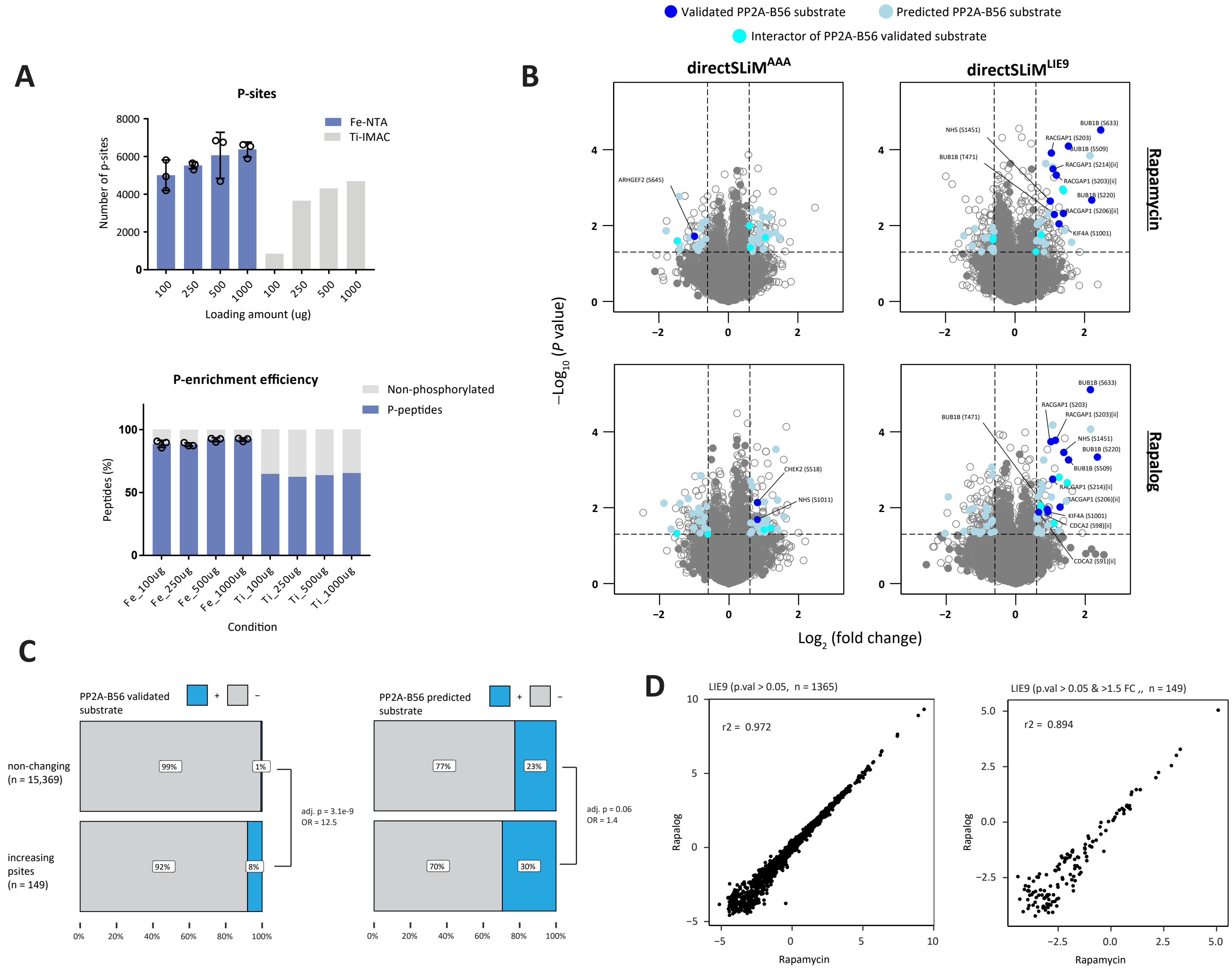

Supplementary Figure 5 (related to Figure 5). A. Comparison of Ti-IMAC beads versus Fe-NTA columns for phosphopeptide enrichment. Different amounts of phosphopeptides from mitotic HeLa-FRT cells were subjected to enrichment by these two methods. Fe-NTA showed the highest number of detected phosphorylation sites with all different loading amounts (top). Fe-NTA also displayed a higher selectivity for phosphopeptides when compared to Ti-IMAC beads (bottom). All loading amounts were processed in triplicate (n=3) for the Fe-NTA (error bars display the standard deviation). For the Ti-IMAC samples were processed once (n=1). **B.** Volcano plot showing upregulated phosphorylation sites after treatment with rapamycin or rapalog in directSLiMs^{LIE9} cells. Solid dots are either validated substrates, predicted substrates or interactors of validated PP2A-B56 substrates (see methods for details). Only hits above a -Log10(P value = 0.05) (Two sided Student's t-test) and >1.5-fold change are color coded. **C.** Assessing enrichment of PP2A-B56 validated (left) and predicted (right) substrates in proteins with increasing phosphorylation sites (p values and odds ratio reported from Fisher's exact test). D. Scatter plot showing correlation of rapamycin and rapalog treatment in all changing sites (left) and in the top changing sites (right) in directSLiMLIE9 cell line. Source data are provided as a Source Data file.

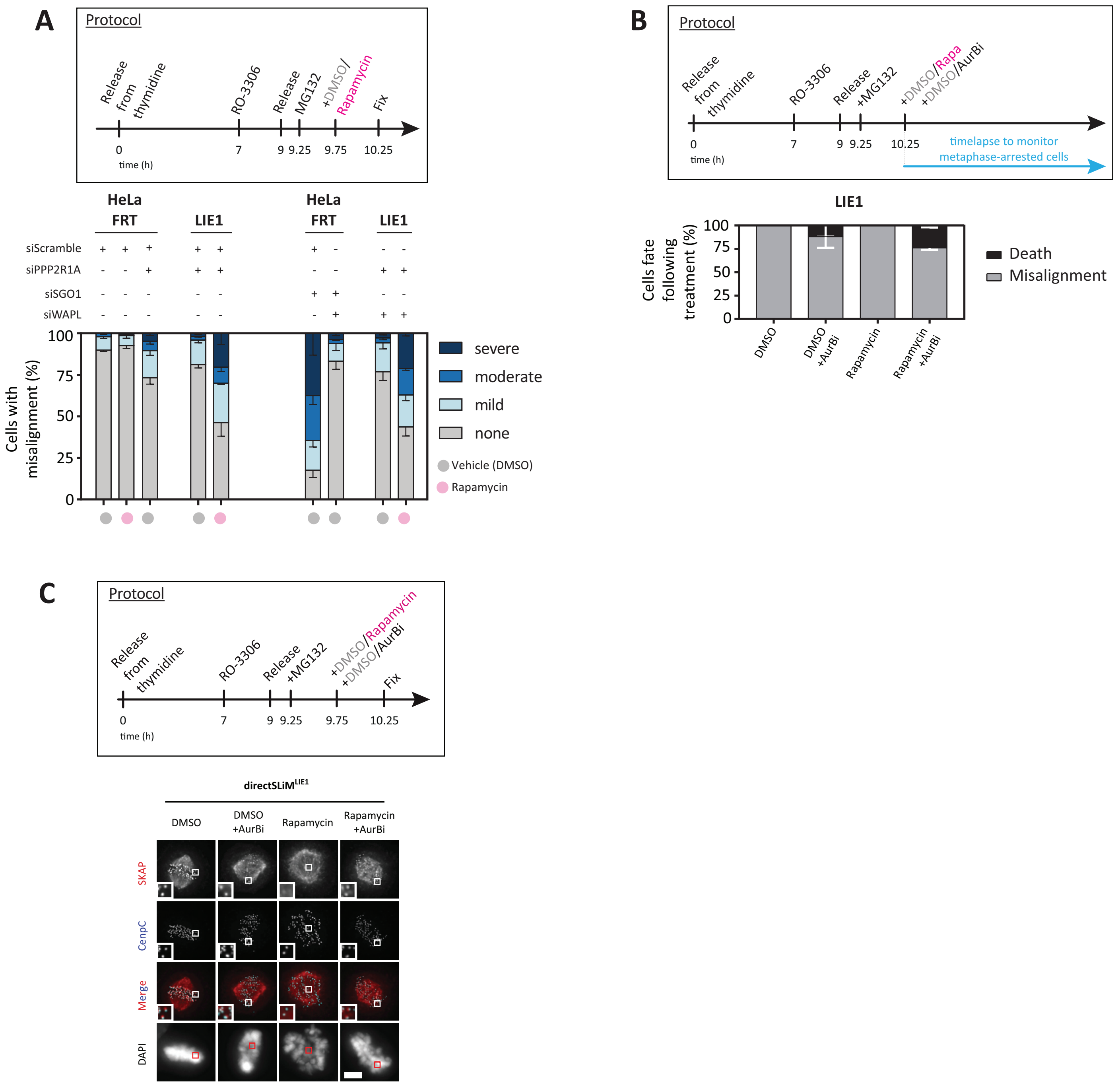

Supplementary Figure 6 (related to Figure 6). Evaluating the effects on chromosome alignment in HeLa FRT cells with/without directSLiM^{LIE1} expression and treated vehicle/rapamycin and indicated siRNAs (A) or ZM-447439 (B-C). **A.** Top panel: protocol used to visualise chromosome alignment in fixed samples (see Materials and Methods for details). Bottom panel: graph showing mean frequencies of chromosome misalignment (±SEM) of 3 experiments, 100 cell quantified per condition per experiment. Individual data points of the bar charts are provided in Source Data file. **B.** Top panel: protocol used to visualise chromosome alignment in live samples (see Materials and Methods for details). Bottom panel: graph showing mean frequencies of the cell fates following treatment (±SEM) of 2 experiments, 50 cells quantified per condition per experiment. Individual data points of the bar charts are provided in Source Data file. Note that those cells experiencing misalignment after treatment are reported in Figure 6F. **C.** Top panel: protocol used to visualise chromosome alignment in fixed samples (see Materials and Methods for details). Bottom panel: Example immunofluorescence images from figure 6I. Scalebar: 5µm. Source data are provided as a Source Data file.

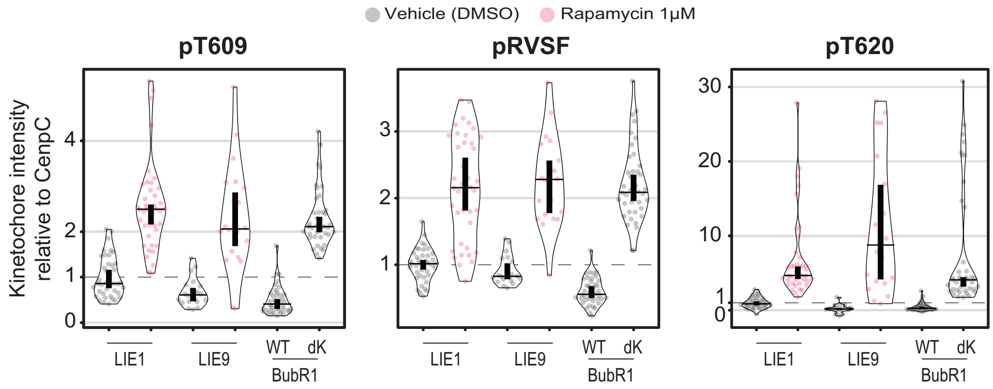

Supplementary Figure 7. Levels of Bub1-pT609, Knl1-pRVSF and BubR1-pT620 at unattached kinetochores, in nocodazole-arrested HeLa FRT cells expressing either directSLiMs$^{LIE1/LIE9}$ or BubR1$^{WT/dK}$, where dK is a BubR1 SLiM mutation that prevents recruitment of PP2A-B56. Cells were treated with vehicle or rapamycin for 20min. Kinetochore intensities a relative to the LIE1 DMSO control. Data from 40 cells, 4 experiments for directSLiM^{LIE1} and BubR1$^{WT/dK}$ and from 20 cells, 2 experiments for directSLiM^{LIE9}. Source data are provided as a Source Data file. Data information: Violin plots show the distributions of kinetochore intensities between cells. For each violin plot, each dot represents an individual cell, the horizontal line represents the median and the vertical one the 95% CI of the median, which can be used for statistical comparison of different conditions (see Materials and Methods).
