# Supplementary material for: A chemical-genetic system to rapidly inhibit the PP2A-B56 phosphatase reveals a role at metaphase kinetochores
Source: Nat Commun. 2025 Mar 29;16:3069. doi: 10.1038/s41467-025-58185-8 (PMC11954910; doi:10.1038/s41467-025-58185-8)
Supplement: Supplementary file 2 — Description of Additional Supplementary Files [file 41467_2025_58185_MOESM2_ESM.pdf]

Description of Additional Supplementary Files

Supplementary Data 1. Results of phosphoproteomics experiment on directSLiM^{LIE1} cells. Contains all data relevant to Figure 2. It also shows if phosphorylation sites have been previously reported as PP2A-B56 substrates according to the database reported in Smith et al (2019) and if they had been previously detected in the screen by Kruse et al (2020).

Supplementary Data 2. Results of phosphoproteomics experiment on directSLiM^{LIE9} cells. Contains all data relevant to Figure 5. It also shows if phosphorylation sites had been previously reported as PP2A-B56 substrates according to the database reported in Smith et al (2019) and if they had been previously detected in the screen by Kruse et al (2020).

Supplementary Data 3. Summary of potential substrates of PP2A-B56 detected in either the directSLiM^{LIE1} and directSLiM^{LIE9} cells. Contains various information of potential PP2A-B56 substrates and the percentages of pSer, pThr, pTyr, Proline directed, and non-Proline directed sites.

Supplementary Movie 1 – Example of live-cell imaging of misalignments during a metaphase arrest in HeLa FRT directSLiM^{AAA} cells treated with DMSO. See Figures 6C-D and Methods for details.

Supplementary Movie 2 – Example of live-cell imaging of misalignments during a metaphase arrest in HeLa FRT directSLiM^{AAA} cells treated with rapamycin. See Figures 6C-D and Methods for details.

Supplementary Movie 3 – Example of live-cell imaging of misalignments during a metaphase arrest in HeLa FRT directSLiM^{LIE1} cells treated with DMSO. See Figures 6C-D and Methods for details.

Supplementary Movie 4 – Example of live-cell imaging of misalignments during a metaphase arrest in HeLa FRT directSLiM^{LIE1} cells treated with rapamycin. See Figures 6C-D and Methods for details.

Supplementary Movie 5 – Example of live-cell imaging of misalignments during a metaphase arrest in HeLa FRT directSLiM^{LIE9} cells treated with DMSO. See Figures 6C-D and Methods for details.

Supplementary Movie 6 – Example of live-cell imaging of misalignments during a metaphase arrest in HeLa FRT directSLiM^{LIE9} cells treated with rapamycin. See Figures 6C-D and Methods for details.

Supplementary Movie 7 – Example of live-cell imaging of misalignments during a metaphase arrest in HeLa FRT directSLiM^{LIE1} cells treated with DMSO. See Figures 6F-G and Methods for details.

Supplementary Movie 8 – Example of live-cell imaging of misalignments during a metaphase arrest in HeLa FRT directSLiM^{LIE1} cells treated with DMSO and rapamycin. See Figures 6F-G and Methods for details.

Supplementary Movie 9 – Example of live-cell imaging of misalignments during a metaphase arrest in HeLa FRT directSLiM^{LIE1} cells treated with DMSO and the AurB inhibitor ZM-447439. See Figures 6F-G and Methods for details.

Supplementary Movie 10 – Example of live-cell imaging of misalignments during a metaphase arrest in HeLa FRT directSLiM^{LIE1} cells treated with rapamycin and the AurB inhibitor ZM-447439. See Figures 6F-G and Methods for details.
