# Supplementary material for: A chemical-genetic system to rapidly inhibit the PP2A-B56 phosphatase reveals a role at metaphase kinetochores
Source: Nat Commun. 2025 Mar 29;16:3069. doi: 10.1038/s41467-025-58185-8 (PMC11954910; doi:10.1038/s41467-025-58185-8)
Supplement: Supplementary file 16 — Reporting Summary [file 41467_2025_58185_MOESM16_ESM.pdf]

nature portfolio

Corresponding author(s): Adrian Saurin

Last updated by author(s): Feb 18, 2025

Reporting Summary

Nature Portfolio wishes to improve the reproducibility of the work that we publish. This form provides structure for consistency and transparency in reporting. For further information on Nature Portfolio policies, see our Editorial Policies and the Editorial Policy Checklist.

Statistics

For all statistical analyses, confirm that the following items are present in the figure legend, table legend, main text, or Methods section.

n/a	Confirmed	
☐	☒	The exact sample size (n) for each experimental group/condition, given as a discrete number and unit of measurement
☐	☒	A statement on whether measurements were taken from distinct samples or whether the same sample was measured repeatedly
☐	☒	The statistical test(s) used AND whether they are one- or two-sided<br>*Only common tests should be described solely by name; describe more complex techniques in the Methods section.*
☐	☒	A description of all covariates tested
☐	☒	A description of any assumptions or corrections, such as tests of normality and adjustment for multiple comparisons
☐	☒	A full description of the statistical parameters including central tendency (e.g. means) or other basic estimates (e.g. regression coefficient) AND variation (e.g. standard deviation) or associated estimates of uncertainty (e.g. confidence intervals)
☐	☒	For null hypothesis testing, the test statistic (e.g. F, t, r) with confidence intervals, effect sizes, degrees of freedom and P value noted<br>*Give P values as exact values whenever suitable.*
☒	☐	For Bayesian analysis, information on the choice of priors and Markov chain Monte Carlo settings
☒	☐	For hierarchical and complex designs, identification of the appropriate level for tests and full reporting of outcomes
☒	☐	Estimates of effect sizes (e.g. Cohen's d, Pearson's r), indicating how they were calculated

Our web collection on statistics for biologists contains articles on many of the points above.

Software and code

Policy information about availability of computer code

Data collection	Thermo Scientific Xcalibur 4.5.445.18 was used to collect MS data. Softworx and Nikon NIS-Elements Software (version 5.42.06) were used to collect imaging data.
Data analysis	Database search of mass spectrometry data was done using MaxQuant 2.4.9.0. Data filtering, statistical analysis and data visualization was done using a combination of Perseus 2.0.11 and R Programming Language (4.4.1). FiJi was used to measure intensity of proteins and phosphosites at the kinetochores. Prism 7 and PlotsOfData (https://huygens.science.uva.nl/PlotsOfData/) were used to represent measures from immunofluorescence and live-cell imaging.

For manuscripts utilizing custom algorithms or software that are central to the research but not yet described in published literature, software must be made available to editors and reviewers. We strongly encourage code deposition in a community repository (e.g. GitHub). See the Nature Portfolio guidelines for submitting code & software for further information.

Data

Policy information about availability of data

All manuscripts must include a data availability statement. This statement should provide the following information, where applicable:

- Accession codes, unique identifiers, or web links for publicly available datasets
- A description of any restrictions on data availability
- For clinical datasets or third party data, please ensure that the statement adheres to our policy

All raw data from this study are available within the article, its Supplementary Figures, its Supplementary Data or on an online repository. All uncropped western

blots and the raw data from all immunofluorescence quantification and live-cell imaging experiments are provided as a Source Data file. Phosphoproteomic data is presented in Supplementary Data 1-3 and has also been deposited in ProteomeXchange Consortium via the PRIDE partner repository with the dataset identifier PXD056390 [http://www.ebi.ac.uk/pride/archive/projects/PXD056390].

Research involving human participants, their data, or biological material

Policy information about studies with human participants or human data. See also policy information about sex, gender (identity/presentation), and sexual orientation and race, ethnicity and racism.

Reporting on sex and gender	*Use the terms sex (biological attribute) and gender (shaped by social and cultural circumstances) carefully in order to avoid confusing both terms. Indicate if findings apply to only one sex or gender; describe whether sex and gender were considered in study design; whether sex and/or gender was determined based on self-reporting or assigned and methods used. Provide in the source data disaggregated sex and gender data, where this information has been collected, and if consent has been obtained for sharing of individual-level data; provide overall numbers in this Reporting Summary. Please state if this information has not been collected. Report sex- and gender-based analyses where performed, justify reasons for lack of sex- and gender-based analysis.*
Reporting on race, ethnicity, or other socially relevant groupings	*Please specify the socially constructed or socially relevant categorization variable(s) used in your manuscript and explain why they were used. Please note that such variables should not be used as proxies for other socially constructed/relevant variables (for example, race or ethnicity should not be used as a proxy for socioeconomic status). Provide clear definitions of the relevant terms used, how they were provided (by the participants/respondents, the researchers, or third parties), and the method(s) used to classify people into the different categories (e.g. self-report, census or administrative data, social media data, etc.) Please provide details about how you controlled for confounding variables in your analyses.*
Population characteristics	*Describe the covariate-relevant population characteristics of the human research participants (e.g. age, genotypic information, past and current diagnosis and treatment categories). If you filled out the behavioural & social sciences study design questions and have nothing to add here, write "See above."*
Recruitment	*Describe how participants were recruited. Outline any potential self-selection bias or other biases that may be present and how these are likely to impact results.*
Ethics oversight	*Identify the organization(s) that approved the study protocol.*

Note that full information on the approval of the study protocol must also be provided in the manuscript.

Field-specific reporting

Please select the one below that is the best fit for your research. If you are not sure, read the appropriate sections before making your selection.

☒ Life sciences ☐ Behavioural & social sciences ☐ Ecological, evolutionary & environmental sciences

For a reference copy of the document with all sections, see nature.com/documents/nr-reporting-summary-flat.pdf

Life sciences study design

All studies must disclose on these points even when the disclosure is negative.

Sample size	For measurement of kinetochore proteins, 10-20 mitotic cells per condition per experiment were used. To score the percentages of mitotic cell fates and mitotic durations, 17-50 cells per conditions were analysed. For percentages of misalignments (fixed assays), 100 cells per condition per experiment.
Data exclusions	One of the LIE1-DMSO-30' control replicates (Figure 2) clearly behaved as an outlier after Principal Components Analysis and Pearson Correlation Coefficient assessment (see Source Data file), hence it was excluded from further downstream analysis. With the exception of this condition (n=2), all other conditions had three biological replicates (n=3).
Replication	Each experiment was conducted with several biological replicates, with the exact number specified in the respective figure legend. To ensure the reproducibility of the findings, several measures were implemented, and key results were validated through complementary experimental approaches.
Randomization	Randomization not relevant, since this study was performed by using cell lines expressing different constructs.
Blinding	The investigators were not blinded to group allocation during the collection of the data. To collect immunofluorescence images, the investigators manually selected mitotic cells expressing high levels of FLAG-PPP2R1A, as stated in Methods. Analysis of mitotic cell fate was performed by manually selecting cells that entered into mitosis during the timelapse and prior to inspection of their cell fate after mitotic entry, so biases are unlikely. Analysis of misalignment timings was performed by manually selecting cells that were arrested in metaphase prior to DMSO/Rapamycin treatment and prior to inspection of their misalignment timings, so biases are unlikely.

Reporting for specific materials, systems and methods

We require information from authors about some types of materials, experimental systems and methods used in many studies. Here, indicate whether each material, system or method listed is relevant to your study. If you are not sure if a list item applies to your research, read the appropriate section before selecting a response.

Materials & experimental systems

n/a	Involved in the study
[ ]	[x] Antibodies
[ ]	[x] Eukaryotic cell lines
[x]	[ ] Palaeontology and archaeology
[x]	[ ] Animals and other organisms
[x]	[ ] Clinical data
[x]	[ ] Dual use research of concern
[x]	[ ] Plants

Methods

n/a	Involved in the study
[x]	[ ] ChIP-seq
[x]	[ ] Flow cytometry
[x]	[ ] MRI-based neuroimaging

Antibodies

Antibodies used

Primary:
1. guinea pig anti CENP-C (PD030 from Caltag + Medsystems)
2. rabbit anti-BUB1 (A300-373A from Bethyl Laboratories)
3. rabbit anti-BUBR1 (A300-386A from Bethyl Laboratories)
4. rabbit anti-KNL1 (ab70537 from abcam)
5. mouse anti-FLAG(M2) (F3165-.2MG from Sigma)
6. mouse anti-B56a (BD Biosciences 610615)
7. mouse anti-B56g (Santa Cruz Biotechnology sc-374379)
8. mouse antiB56d (Santa Cruz Biotechnology sc-271363)
9. rabbit B56e (Aviva ARP56694-P050)
10. mouse anti-PPP2CA (EMD Millipore 05-421)
11. rabbit anti-PPP2R1A (Genetex GTX102206)
12. rabbit anti-GEF-H1 (Abcam 155785)
13. rabbit anti-CDCA2 (Repoman, Sigma HPA030049)
14. rabbit anti-Actin (Sigma A2066)
15. rabbit anti-pMELT-KNL1 - directed against phospho-Thr 943 and -Thr 1155 of human KNL1 (Nijenhuis et al., Nat Cell Biol 2014) (gift from G. Kops, Hubrecht, NL).
16. rabbit anti-pRVSF-KNL1 - raised against phospho-Ser 60 of human KNL1 (using the peptide C-CKKNSRRV[pS]FADTIK, custom raised by Biomatik).
17. rabbit anti-BUBR1-pT620 - raised against phospho-Thr 620 of human BUBR1 using the peptide CAARFVS[pT]PFHE (custom raised by Moravian) (Cordeiro et al. JCB 2020).
18. rabbit anti-BUB1-pT609 - raised against phospho-Thr 609 of human BUB1 using the peptide CAQLAS[pT]PFHKLPVES (custom raised by Biomatik) (Cordeiro et al. JCB 2020).
19. The rabbit anti-BUB1-pT461 – raised against phospho-Thr 461 of human BUB1 (Qian et al., Mol Cell 2017) (gift from M. Bollen, Leuven, BE)
20. mouse anti-BUBR1(8G1) (05-898 from Millipore)
21. rabbit anti-SKAP(KNSTRN) (HPA042027 from Atlas Antibodies)
22. rabbit anti-HEC1-pSer55 (GTX70017 from Genetex)
23. rabbit anti-mCherry (GTX128508 from Genetex)
24. mouse anti-Tubulin (T5168 from Sigma)

Secondary for immunofluorescence (highly-cross absorbed):
• goat anti-chicken Alexa Fluor 488 (A-11039 - Thermo Fisher)
• goat anti-rabbit Alexa Fluor 568 (A-11036 - Thermo Fisher)
• goat anti-mouse Alexa Fluor 488 (A11029- Thermo Fisher)
• goat anti-mouse Alexa Fluor 568 (A-11031- Thermo Fisher)
• goat anti-guinea pig Alexa Fluor 647 (A-21450- Thermo Fisher)
• donkey anti-rabbit Alexa Fluor 647 (A-31573- Thermo Fisher)
• donkey anti-mouse Alexa Fluor 647 (A31571- Thermo Fisher)

Secondary for Western Blot:
• mouse IgG HRP conjugate (Bio-Rad 170–6516)
• goat anti-rabbit IgG HRP conjugate (BioRad 1706515)
• IRDye® 800CW goat anti-mouse (LICORbio, 926-32210)
• IRDye 800CW donkey anti-rabbit (LICORbio, 926-32213)
• goat anti-mouse light chain specific HRP-conjugated (Sigma, AP200P, 1:1000).

Validation

1. Guinea pig anti-CENP-C (PD030, Caltag + Medsystems-MBL) – manufacturer states that the product is suitable for the following applications: ICC, IP and WB. Species reactivity: human. Manufacturer provides numerous references that have used this antibody.

2. Rabbit anti-BUB1 (A300-373A, Bethyl Laboratories) - manufacturer states that the product is suitable for the following applications: WB, IP and IHC. Species reactivity: human. Manufacturer shows specificity by increased levels in mitosis, by IP-western and provides numerous references that have used this antibody.
3. Rabbit anti-BUBr1 (A300-386A, Bethyl Laboratories) - manufacturer states that the product is suitable for the following applications: WB and IP. Species reactivity: human, mouse & rat. Predicted species reactivity: panda, orangutan, monkey and gorilla. Manufacturer shows specificity by IP-western and provides numerous references that have used this antibody.
4. Rabbit anti-KNL1 (ab750537, Abcam) – manufacturer states that the product is suitable for the following applications: IP and WB. Species reactivity: human. Predicted species reactivity: chimpanzee, gorilla, orangutan and rhesus monkey. Manufacturer shows specificity by IP-western and provides numerous references that have used this antibody.
5. Mouse anti-FLAG (M2) (F3165, Sigma) – manufacturer states that the monoclonal antibody recognises the FLAG sequence at the N-terminus, Met N-terminus, and C-terminus. The antibody is also able to recognise FLAG at an internal site. Immunogen: DYKDDDDK. The product is suitable for the following applications: WB, IP, IHC, IF, ELISA, EIA, CHIP, EM, FC and supershift assays. Manufacturer provides numerous references that have used this antibody.
6. Mouse anti-B56a (610615, BD Biosciences) - - manufacturer states that the product is suitable for the following applications: WB and IHC. Species reactivity: rat, human, mouse, dog, chicken and frog. We previously validated this antibody by siRNA-knockdown of B56a followed by WB (Vallardi et al., eLife 2019; doi: 10.7554/eLife.42619). Manufacturer provides numerous references that have used this antibody.
7. Mouse anti-B56g (sc-374379, Santa Cruz) - - manufacturer states that the product is suitable for the following applications: WB and IP, IF and ELISA. Species reactivity: mouse, rat and human. Manufacturer provides several references that have used this antibody.
8. Raised against aa 509-573 mapping near the C-term of PP2A-B56-d of human origin; recommended for detection of PP2A-B56-d of mouse, rat and human origin by WB, IP, IF and ELISA; PMIDs of relevant citations: 35878017, 30829571
9. The immunogen is a synthetic peptide corresponding to the following mouse B56e region: SCNIFRTLPPSDSNEFDPEEDEPTLEASWPHLQLVYEFFIRFLESQEFQP; predicted species reactivity: Human, Mouse, Rat, Cow, Dog, Goat, Guinea Pig, Horse, Rabbit, Zebrafish; application: WB; PMIDs of relevant citations: 30829571
10. The immunogen is a 16 residue synthetic peptide (C-RGEPHVTRRTPDYFL) corresponding to amino acids 295-309 of the 36 kDa catalytic subunit of human protein phosphatase 2A (PP2A). Clone 1D6; species reactivity: human, rat, mouse, bovine, rabbit, xenopus, S. cerevisiae; applications: IC, IP, WB
11. The manufacturer stated that the immunogen is a recombinant protein encompassing a sequence within the center region of human PPP2R1A. The exact sequence is proprietary of the manufacturer; reactivity: human, mouse; applications: WB, ICC/IF, IHC-P, IP; manufacturer provides numerous references that have used this antibody
12. Immunogen corresponding to Recombinant Fragment Protein within human ARHGEF2 aa 650 to C-term; suitable for IP, WB, IHC-P, ICC/IF and reacts with human, mouse samples; cited in 26 publications.
13. Immunogen sequence: IKCERKDDFLGAAEGKLQCNRLMPNSQKDCHCLGDVLIENTKESKSQSEDLGRKPMESSSVVSCRDRKDRRRSMCYSDGRSLHLEKNGNHTPSS; species reactivity: human; applications: IF, IC; manufacturer provides numerous references that have used this antibody
14. Species reactivity: wide range, vertebrates, human, slime mold, amoeba, chicken; suitable for IC, IF, WB; manufacturer provides numerous references that have used this antibody
15. Directed against phospho-Thr 943 and -Thr 1155 of human KNL1 (Nijenhuis et al., Nat Cell Biol 2014) (gift from G. Kops, Hubrecht, NL)
16. Custom antibody (Biomatik) raised against phospho-Ser 60 of human KNL1 (using the peptide C-CKKNSRRV[pS]FADTIK)
17. Custom antibody (Moravian) raised against phospho-Thr 620 of human BUBR1 using the peptide CAARFVS[pT]PFHE (Cordeiro et al. JCB 2020).
18. Custom antibody (Biomatik) raised against phospho-Thr 609 of human BUB1 using the peptide CAQLAS[pT]PFHKLPVES (Cordeiro et al. JCB 2020).
19. Raised against phospho-Thr 461 of human BUB1 (Qian et al., Mol Cell 2017) (gift from M. Bollen, Leuven, BE)
20. The immunogen is a 6His-tagged fusion protein corresponding to full length human BubR1. Species reactivity: human. Manufacturer states that the product is suitable for Immunoblot analysis, immunoprecipitation and immunocytochemistry and provides numerous references that have used this antibody.
21. Antigen sequence: TVYSLQPPSALSGGQPADTQTRATSKSLLPVRSKEVDVSKQLHSGGPENDVTKITKLRRENGQMKATDTATRRNVRKGYKPLSKQ. The manufacturer verified species reactivity with human. The manufacturer states the antibody is suitable for immunofluorescence and immunohistochemistry and provides numerous references that have used this antibody
22. The immunogen is a Carrier-protein conjugated synthetic peptide surrounding phospho Ser55 of human Hec1 (the manufacturer states the exact sequence is proprietary). Species reactivity: human, mouse. The manufacturer states the antibody is suitable for WB and provides numerous references that have used this antibody.
23. Rabbit anti-mCherry (GTX128508, Genetex) - manufacturer states that the product was raised against full length mCherry recombinant protein and that it is suitable for the following applications: WB, ICC/IF, IHC-Fr, IHC-Wm, IP, PLA. Manufacturer shows specificity by WB and IP-WB and provides numerous references that have used this antibody.
24. Mouse anti-Tubulin (T5168, Sigma) - species reactivity: mouse, chicken, Chlamydomonas, African green monkey, human, rat, bovine, sea urchin and kangaroo rat. The manufacturer states that the product is suitable for the following applications: IF, radioimmunoassay, and WB, and provides several references that have used this antibody.

Eukaryotic cell lines

Policy information about cell lines and Sex and Gender in Research

Cell line source(s)	HeLa Flp-in cells (gift from S Taylor, University of Manchester, UK) and Phoenix Ampo 293 cells (ATCC)
Authentication	STR profiling (Eurofins)
Mycoplasma contamination	Cells were screened to ensure a mycoplasma-free culture

Commonly misidentified lines (See ICLAC register)	No commonly misidentified cell lines were used in this study

Plants

Seed stocks	*Report on the source of all seed stocks or other plant material used. If applicable, state the seed stock centre and catalogue number. If plant specimens were collected from the field, describe the collection location, date and sampling procedures.*
Novel plant genotypes	*Describe the methods by which all novel plant genotypes were produced. This includes those generated by transgenic approaches, gene editing, chemical/radiation-based mutagenesis and hybridization. For transgenic lines, describe the transformation method, the number of independent lines analyzed and the generation upon which experiments were performed. For gene-edited lines, describe the editor used, the endogenous sequence targeted for editing, the targeting guide RNA sequence (if applicable) and how the editor was applied.*
Authentication	*Describe any authentication procedures for each seed stock used or novel genotype generated. Describe any experiments used to assess the effect of a mutation and, where applicable, how potential secondary effects (e.g. second site T-DNA insertions, mosiacism, off-target gene editing) were examined.*
